# The association of dietary iron intake and serum iron with fecal incontinence: results from NHANES 2007–2010

**DOI:** 10.3389/fnut.2025.1598172

**Published:** 2025-06-19

**Authors:** Tingting Li, Jingxuan Cui, Yan Zhou, Qionglu Yao, Lijun Cai

**Affiliations:** ^1^The First School of Clinical Medicine, Zhejiang Chinese Medical University, Hangzhou, China; ^2^Department of Gastroenterology, The First Affiliated Hospital of Zhejiang Chinese Medical University (Zhejiang Provincial Hospital of Chinese Medicine), Hangzhou, China

**Keywords:** iron intake, serum iron, fecal incontinence, NHANES, ferritin

## Abstract

**Background:**

The relationship between iron and fecal incontinence (FI) is unclear. This study aims to explore the association between iron intake and serum iron levels and FI subtypes.

**Methods:**

8,612 adults from the National Health and Nutrition Examination Survey 2007–2010 were included in the study. FI was determined by the Bowel Health Questionnaire. This study corrected for demographic characteristics, chronic diseases and so on.

**Results:**

Compared to quartile 1, quartile 3 of dietary iron was associated with a higher risk of gas gut leakage (OR = 1.35, 95%CI:1.05–1.73), and quartile 4 of serum iron was associated with a lower risk of solid bowel leakage (OR = 0.42, 95%CI: 0.20–0.89). Restricted cubic spline (RCS) models showed an inverted U-shaped association between iron intake and the prevalence of gas gut leakage (P for nonlinear < 0.001). When iron intake is between 13.68 and 21.55 mg/day, the risk of gas gut leakage is significantly increased. However, serum iron was significantly negatively linearly correlated with solid stool leakage. Subgroup analysis suggested that there was heterogeneity in the association between iron and FI in terms of gender and age. The association is stronger in women and people aged 60 to 74 years. In exploratory analysis, higher ferritin levels in women of childbearing age were associated with a lower chance of mucus gut leakage.

**Conclusion:**

Lower serum iron levels and moderate iron intake may be associated with an increased risk of FI in adults, with gender and age differences. Older women may need to increase their iron intake, which may be beneficial in preventing FI. However, the causal relationship still needs to be verified by prospective studies.

## 1 Introduction

Fecal incontinence (FI) is defined as the involuntary loss of solid or liquid feces, gases, or mucus (1, 2). It is a debilitating condition that significantly affects the quality of life. FI includes a range of symptoms, including passive leakage, emergency-related accidents, and incomplete evacuation (3, 4). According to epidemiological research, the prevalence of FI in community adults varies between 1.4 and 19.5% worldwide, and it rises sharply with age (5–7). The risk is more than three times higher in people over the age of 65 than in younger people (5, 8). Despite the clinical and psychosocial burdens, FI continues to be underreported and undertreated due to stigma and diagnostic challenge (3). Although some progress has been made in interventions such as biofeedback therapy and sacral nerve stimulation, about 30 to 40% of patients do not respond well to existing treatments, and the long-term efficacy is not stable (1, 9). Recent high-impact reviews have highlighted the multiple factors of FI, including pelvic floor dysfunction, visceral hypersensitivity, and altered bowel motility (1, 3, 8). However, gaps remain in understanding modifiable nutritional effects.

Iron is an essential trace element in the human body, and moderate intake can promote intestinal cell growth, maintain immune and digestive functions, but excessive intake can cause oxidative damage and imbalance of microflora (10–13). According to previous studies, intestinal flora disorders or iron deficiency are linked to an imbalance in iron metabolism, which can lead to intestinal inflammation and even cancer (14–16). Notably, iron excess may affect bowel motility by causing diarrhea, which is a known risk factor for fecal incontinence (17, 18). These findings suggest a two-way relationship between iron and gut health, and further research is needed.

Dietary iron intake and serum iron work together to regulate iron homeostasis. Non-heme iron (plant source) and heme iron (animal source) exhibit different absorption efficiencies mediated by duodenal ferrotransporter expression under the control of the main iron-regulating hormone, hepcidin (19). Studies have found that high dietary iron intake may induce mucositis by inhibiting gut microbiota diversity (12). High serum iron levels appear to be associated with a reduced risk of colon cancer, but not significantly (20). Recent studies have found an association between unsafe diets, trace elements, and heavy metals in the blood and FI (21–24). However, the relationship of dietary iron and serum iron with FI risk has not been systematically studied to date.

Through a cross-sectional population cohort analysis of NHANE, this study investigates the connection between dietary iron intake, serum iron levels, and FI risk. The findings of this study suggest that dietary iron intake should be changed to prevent fecal incontinence. This can help identify high-risk individuals among those with abnormal iron metabolism. In addition, optimizing serum iron levels may be more beneficial for FI prophylaxis.

## 2 Materials and methods

### 2.1 Data sources and study populations

Publicly available data came from the National Health and Nutrition Survey (NHANES). Participants were invited to a Mobile Examination Centre (MEC) for a series of laboratory tests. Blood, urine, and other biological samples were collected as part of these tests. Laboratory data was collected by professionals in a controlled environment through standardized methods, ensuring data accuracy and consistency. The questionnaire was collected at the respondent’s home through the computer-assisted personal interviewing (CAPI) system by trained interviewers. They all voluntarily signed a written agreement. The data was available on the CDC website.^1^

**Figure 1:**
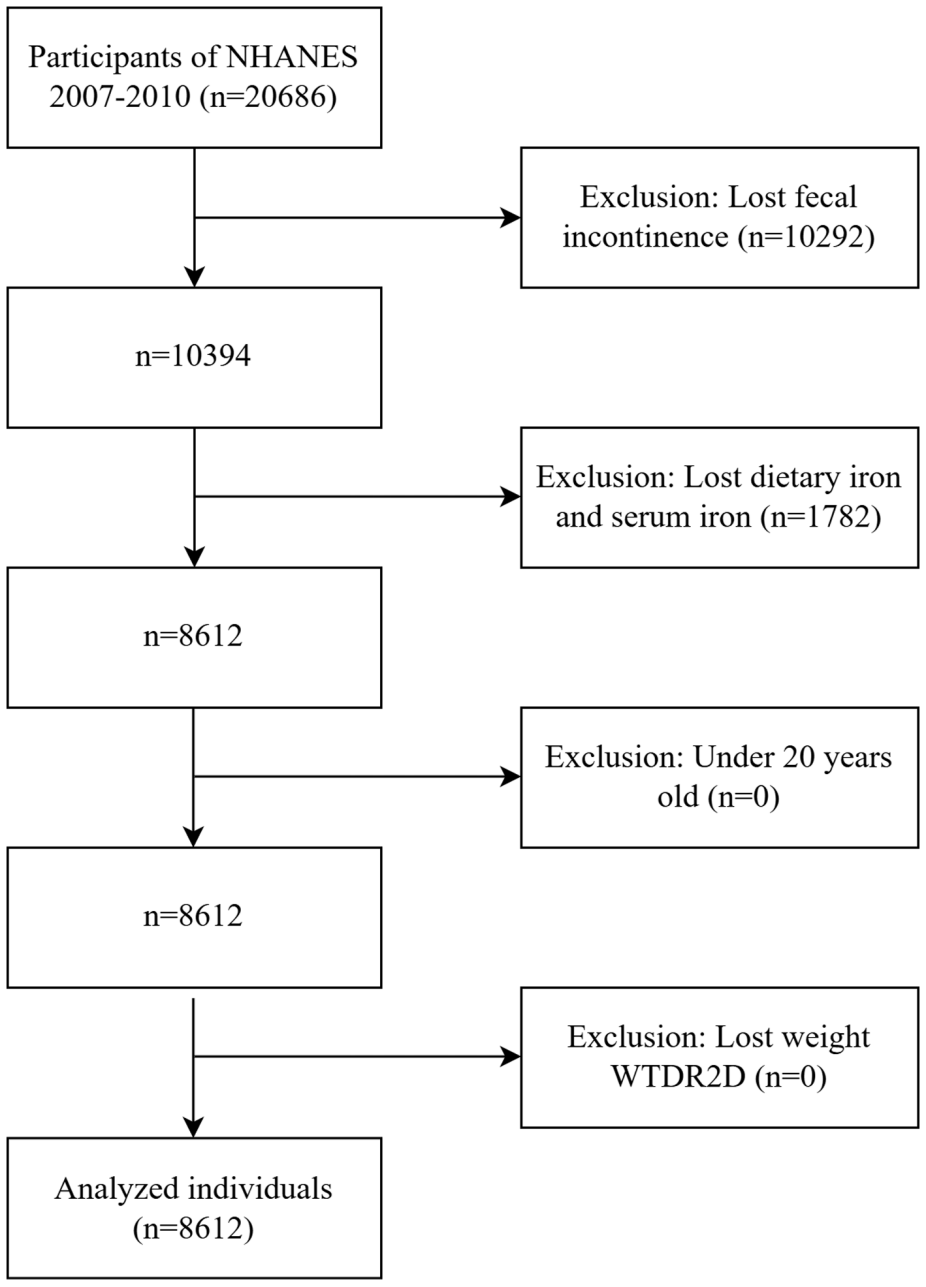
The process of inclusion and exclusion of research subjects.

The collection of the Bowel Health Questionnaire (which includes questions related to FI) was first implemented in the 2005–2006 cycle and stopped in the 2009–2010 cycle. The 2007–2010 cycle updated the experimental assessment method for serum iron. In order to reduce measurement variability, we selected the 2007–2010 period for data analysis. NHANES 2007–2010 had a total of 20,686 participants. The study included individuals aged ≥ 20 years who had complete dietary iron intake (24-h dietary recall), serum iron data, and the Fecal Incontinence Questionnaire (via the Digestive Health module). The data were weighted according to the sampling weights provided by NHANES. Participants with missing weights were excluded. A total of 8,612 participants were included, 4,184 males and 4,428 females. Figure 1 depicts a flowchart of the inclusion process.

### 2.2 Fecal incontinence

Fecal incontinence (FI) includes gas leakage, mucus leakage, liquid leakage, and solid stool bowel leakage. We collected information about FI through the Bowel Health Questionnaire. In this study, participants who had 1 to 3 or more uncontrolled bowel leakage of gas (mucus/liquid/solid stool) from the intestine in a month were inferred to have a gas (mucus/liquid/solid stool) intestinal leakage. Similarly, the mucus method had been widely used in previous studies (25–27).

### 2.3 Dietary iron and serum iron

NHANES’ dietary data collected detailed dietary intake of participants on the first and second days, respectively. Estimate the amount of energy and nutrients ingested in them by asking about the type and amount of food and drink consumed the day before. Collected through two 24-h food recall interviews. The first interview was conducted at the Mobile Examination Center (MEC), and the second interview was conducted by telephone 3–10 days later. Dietary iron intake was based on the average (mg/day) of two 24-h dietary recalls. Serum iron data were measured by the DcX800 method, a timed endpoint method. Detailed measurement procedures could be found on the NHANES website.

### 2.4 Covariates

Confounding factors that may be linked to FI were included in this study based on prior research. Age, gender, race (Non-Hispanic White, Non-Hispanic Black, and others), level of education (below college, college or above), household income-to-poverty ratio (PIR), and body mass index (BMI) are examples of demographic variables. Overweight is defined as a BMI < 25 kg/m^2^, obesity is defined as a BMI ≥ 30 kg/m^2^, with a BMI of 25–30 kg/m^2^ being normal. Depression, diabetes, hypertension, and lifestyle factors like alcohol and cigarette use were also included. Depression is measured by the Patient Health Questionnaire-9 (PHQ-9). A PHQ-9 score of more than 10 is thought to be significantly associated with depressive status (28). Individuals meeting any of the following criteria were diagnosed with diabetes: (1) physician-confirmed diagnosis; (2) current therapeutic regimen involving oral hypoglycemic agents or exogenous insulin administration; or (3) biochemical evidence demonstrated by either glycated hemoglobin (HbA1c) levels ≥6.5% or fasting plasma glucose concentrations ≥126 mg/dL (7.0 mmol/L). Participants were divided into recent smokers, former smokers and never smokers based on whether they had a history of smoking and whether they were smoking now. Alcohol use was determined by whether people had drunk at least 12 glasses of alcohol in their lives. The definitions of each variable are detailed in Supplementary Table S1.

### 2.5 Statistical analysis

Weighted mean ±SD, frequency, and weighted percentages were used to describe quantitative and categorical variables, respectively. Logistic regression models were established to analyze the relationship between dietary iron and serum iron and FI (gas gut leakage, mucus gut leakage, liquid intestinal leakage, solid intestinal leakage). In addition, with cut-off points based on the distribution in participants, the levels of dietary and serum iron were stratified into quartiles (Q1, <25th; Q2, 25th–50th; Q3, 50th–75th; Q4, >75th percentile). The Model 1 adjusted for age and gender; Model 2 adjusted for race, education, and PIR from Model 1; Model 3 incorporated BMI, smoking, alcohol consumption, high blood pressure, diabetes, and depression on top of Model 2. *p* values below 0.05 were regarded as statistically significant. *P* for trend less than 0.05 was considered to have a significant trend. To further verify whether there was a dose–response relationship between iron and FI, we used a restricted cubic spline (RCS) to fit a nonlinear relationship. Three nodes (5th, 50th, 95th) were set up, and the OR value and 95% CI were calculated with the median as the reference value. A likelihood ratio test (*P* for nonlinear) of less than 0.05 was considered to have a nonlinear relationship. The above data analysis was performed using R software. Finally, subgroup analysis was used to stratify by gender, age, BMI, etc., to further explore the association heterogeneity between iron and FI. To gain a more complete understanding of the relationship of iron status with FI, we explored the association of other biomarkers of iron homeostasis (ferritin and transferrin receptors) with FI in a subgroup of women of childbearing age.

All analysis incorporated NHANES sampling weights to ensure national representativity. Confounding factors—including age, sex, race/ethnicity, household income, and chronic diseases (diabetes, IBD)—were adjusted for in multivariable logistic regression models rather than excluding participants.

## 3 Results

### 3.1 Baseline characters of participants

A total of 8,612 participants were included in this study from the NHANES 2007–2008 and 2009–2010 cycles. Baseline characteristics of participants are presented in Table 1. Among them, 3,740 people were diagnosed with gas gut leakage, 286 people with mucus gut leakage, 600 people with liquid gut leakage, and 206 people with solid fecal gut leakage. Participants with leaky gut tended to be older than those who did not. According to the age division of the WHO, the average age of participants with solid stool leakage was 60.97 years (in the elderly). The average age of the other types of leaky gut patients was between 45 and 69 years (middle-aged). There were statistically significant differences in age, ethnicity, education level, and serum iron concentration between the solid and non-solid stool leakage groups (all *p* < 0.05). The serum iron concentrations and iron intake of the gaseous, mucous, liquid, and non-leaky groups did not differ significantly. Patients with bowel leakage tended to be middle-aged and older women, non-Mexican-white, and suffer from high blood pressure, diabetes, and depression to a large extent.

**Table 1:** The basic characteristics of the participants in NHANES 2007–2008, 2009–2010.

Characteristic	Bowel leakage of gas			Bowel leakage of mucus			Bowel leakage of liquid			Bowel leakage of solid stool		
	No (*N* = 4,872)	Yes (*N* = 3,740)	*p*	No (*N* = 8,326)	Yes (*N* = 286)	*p*	No (*N* = 8,012)	Yes (*N* = 600)	*p*	No (*N* = 8,406)	Yes (*N* = 206)	*p*
Age [Mean (SD)]	45.62 (16.46)	48.83 (16.51)	<0.001	46.82 (16.55)	54.50 (14.95)	<0.001	46.41 (16.48)	57.09 (14.49)	<0.001	46.78 (16.47)	60.97 (15.00)	<0.001
Gender, *n* (%)			0.001			0.251			0.031			0.107
Male	2,501 (50.5%)	1,683 (44.0%)		4,067 (47.8%)	117 (43.5%)		3,924 (48.1%)	260 (40.8%)		4,104 (47.9%)	80 (37.1%)	
Female	2,371 (49.5%)	2,057 (56.0%)		4,259 (52.2%)	169 (56.5%)		4,088 (51.9%)	340 (59.2%)		4,302 (52.1%)	126 (62.9%)	
Race, *n* (%)			<0.001			0.527			<0.001			0.035
Non-Hispanic White	2,274 (67.4%)	2,095 (75.1%)		4,220 (70.8%)	149 (70.1%)		3,994 (70.3%)	375 (79.7%)		4,246 (70.7%)	123 (78.5%)	
Non-Hispanic Black	995 (12.1%)	523 (8.4%)		1,483 (10.5%)	35 (7.6%)		1,412 (10.5%)	106 (10.0%)		1,484 (10.4%)	34 (11.2%)	
Other	1,603 (20.5%)	1,122 (16.5%)		2,623 (18.6%)	102 (22.3%)		2,606 (19.2%)	119 (10.3%)		2,676 (18.9%)	49 (10.4%)	
Education, *n* (%)			0.696			0.569			0.749			0.006
Below college	2,520 (42.5%)	1,914 (42.0%)		4,267 (42.2%)	167 (45.3%)		4,116 (42.3%)	318 (41.2%)		4,308 (42.0%)	126 (57.5%)	
College or above	2,352 (57.5%)	1,826 (58.0%)		4,059 (57.8%)	119 (54.7%)		3,896 (57.7%)	282 (58.8%)		4,098 (58.0%)	80 (42.5%)	
PIR, *n* (%)			0.440			0.261			0.814			0.223
Not poor	2,746 (67.8%)	2,159 (68.8%)		4,756 (68.4%)	149 (62.1%)		4,585 (68.3%)	320 (67.7%)		4,798 (68.3%)	107 (63.5%)	
Poor	2,126 (32.2%)	1,581 (31.2%)		3,570 (31.6%)	137 (37.9%)		3,427 (31.7%)	280 (32.3%)		3,608 (31.7%)	99 (36.5%)	
Body mass index, *n* (%)			0.051			0.447			0.004			0.938
Normal	1,400 (32.2%)	1,006 (28.5%)		2,330 (30.5%)	76 (31.9%)		2,266 (31.0%)	140 (23.3%)		2,337 (30.5%)	69 (31.6%)	
Obese	1,787 (34.2%)	1,462 (37.4%)		3,130 (35.5%)	119 (39.7%)		2,983 (35.0%)	266 (45.8%)		3,177 (35.6%)	72 (35.4%)	
Overweight	1,685 (33.6%)	1,272 (34.1%)		2,866 (34.0%)	91 (28.4%)		2,763 (34.0%)	194 (30.9%)		2,892 (33.8%)	65 (33.0%)	
Smoke, *n* (%)			0.556			0.383			0.213			0.049
Former smoker	1,253 (25.3%)	995 (25.4%)		2,161 (25.2%)	87 (28.8%)		2,046 (25.0%)	202 (30.2%)		2,169 (25.1%)	79 (36.4%)	
Never smoker	2,577 (53.6%)	1,984 (54.6%)		4,428 (54.3%)	133 (46.7%)		4,302 (54.4%)	259 (49.0%)		4,471 (54.3%)	90 (42.4%)	
Recent smoker	1,042 (21.1%)	761 (20.0%)		1,737 (20.5%)	66 (24.5%)		1,664 (20.6%)	139 (20.8%)		1,766 (20.6%)	37 (21.2%)	
Alcohol, *n* (%)	3,524 (76.9%)	2,690 (76.7%)	0.853	6,011 (76.9%)	203 (76.9%)	1.000	5,782 (77.0%)	432 (75.1%)	0.450	6,082 (77.1%)	132 (66.4%)	0.047
Diabetes, *n* (%)	773 (11.0%)	688 (12.8%)	0.029	1,386 (11.6%)	75 (21.1%)	<0.001	1,300 (11.3%)	161 (19.8%)	<0.001	1,400 (11.6%)	64 (22.5%)	0.001
Hypertension, *n* (%)	1,648 (26.9%)	1,447 (33.2%)	<0.001	2,961 (29.3%)	134 (43.5%)	0.001	2,768 (28.6%)	327 (46.8%)	<0.001	2,981 (29.2%)	114 (56.4%)	<0.001
Depression, *n* (%)	359 (6.2%)	451 (10.2%)	<0.001	744 (7.4%)	66 (26.3%)	<0.001	681 (7.2%)	129 (20.8%)	<0.001	773 (7.9%)	37 (13.1%)	0.029
Dietary iron [Mean (SD)]	15.70 (8.12)	15.37 (7.25)	0.219	15.59 (7.77)	14.46 (6.92)	0.083	15.58 (7.77)	15.05 (7.40)	0.071	15.56 (7.75)	15.06 (7.60)	0.443
Serum iron [Mean (SD)]	15.63 (6.47)	15.31 (6.23)	0.102	15.49 (6.39)	15.35 (5.62)	0.742	15.52 (6.41)	15.03 (5.66)	0.222	15.52 (6.37)	13.84 (5.96)	0.026

### 3.2 Relationship between dietary iron, serum iron, and bowel leakage

The results of the logistic regression for leaky gut, serum iron, and dietary iron are displayed in Table 2. The findings indicated that bowel leakage was related to dietary iron intake (*p* < 0.05). A 35% higher risk of gaseous gut leakage was linked to dietary iron in the third quartile in fully corrected model 3 than to the first quartile (95% CI: 1.05–1.73, *p* = 0.02). A positive correlation between dietary iron intake and the probability of bowel leakage was also observed in the second and fourth quartiles, but no statistical difference was found (*p* > 0.05). There was no significant trend between dietary iron and bowel leakage (*P* for trend >0.05).

**Table 2:** Association of bowel leakage with iron intake and serum iron.

Item	Model 1	Model 2	Model 3
	OR (95%CI) *p*-value	OR (95%CI) *p*-value	OR (95%CI) *p*-value
Bowel leakage of gas			
Iron intake (mg/day)			
Q1 (<9.95)	Ref	Ref	Ref
Q2 (9.95–13.45)	1.08 [0.87, 1.34] 0.491	1.07 [0.86, 1.32] 0.526	1.09 [0.87, 1.37] 0.414
Q3 (13.45–18.36)	**1.35 [1.06, 1.73] 0.018**	**1.33 [1.05, 1.68] 0.022**	**1.35 [1.05, 1.73] 0.021**
Q4 (>18.36)	1.08 [0.87, 1.36] 0.461	1.05 [0.85, 1.31] 0.627	1.08 [0.86, 1.37] 0.476
*P* for trend	0.191	0.295	0.226
Serum iron (umol/L)			
Q1 (<11.1)	Ref	Ref	Ref
Q2 (11.1–14.5)	0.94 [0.78, 1.12] 0.443	0.91 [0.76, 1.09] 0.294	0.92 [0.76, 1.12] 0.379
Q3 (14.5–18.8)	0.98 [0.84, 1.15] 0.777	0.95 [0.81, 1.11] 0.480	0.97 [0.82, 1.13] 0.649
Q4 (>18.8)	0.90 [0.75, 1.09] 0.273	0.86 [0.71, 1.05] 0.127	0.89 [0.73, 1.08] 0.224
*P* for trend	0.331	0.147	0.262
Bowel leakage of mucus			
Iron intake (mg/day)			
Q1 (<9.95)	Ref	Ref	Ref
Q2 (9.95–13.45)	1.17 [0.70, 1.94] 0.535	1.20 [0.73, 1.96] 0.458	1.33 [0.79, 2.26] 0.260
Q3 (13.45–18.36)	1.33 [0.73, 2.42] 0.342	1.37 [0.74, 2.55] 0.296	1.59 [0.83, 3.04] 0.146
Q4 (>18.36)	0.69 [0.37, 1.29] 0.234	0.72 [0.38, 1.34] 0.284	0.83 [0.44, 1.59] 0.552
*P* for trend	0.394	0.481	0.854
Serum iron (umol/L)			
Q1 (<11.1)	Ref	Ref	Ref
Q2 (11.1–14.5)	1.58 [0.94, 2.67] 0.082	1.60 [0.95, 2.71] 0.077	1.74 [1.01, 2.99] 0.045
Q3 (14.5–18.8)	1.40 [0.86, 2.29] 0.172	1.41 [0.86, 2.32] 0.169	1.50 [0.90, 2.50] 0.111
Q4 (>18.8)	1.29 [0.78, 2.13] 0.303	1.31 [0.79, 2.15] 0.279	1.39 [0.85, 2.28] 0.178
*P* for trend	0.443	0.420	0.290
Bowel leakage of liquid			
Iron intake (mg/day)			
Q1 (<9.95)	Ref	Ref	Ref
Q2 (9.95–13.45)	1.12 [0.73, 1.72] 0.592	1.13 [0.73, 1.75] 0.566	1.23 [0.77, 1.96] 0.364
Q3 (13.45–18.36)	1.36 [0.94, 1.96] 0.097	1.36 [0.93, 1.99] 0.106	1.51 [1.00, 2.26] 0.049
Q4 (>18.36)	0.97 [0.65, 1.42] 0.854	0.96 [0.64, 1.44] 0.843	1.09 [0.71, 1.68] 0.681
*P* for trend	0.779	0.824	0.353
Serum iron (umol/L)			
Q1 (<11.1)	Ref	Ref	Ref
Q2 (11.1–14.5)	1.08 [0.77, 1.51] 0.653	1.07 [0.77, 1.51] 0.664	1.13 [0.79, 1.60] 0.473
Q3 (14.5–18.8)	1.13 [0.75, 1.71] 0.549	1.13 [0.74, 1.72] 0.563	1.22 [0.78, 1.89] 0.363
Q4 (>18.8)	0.93 [0.59, 1.47] 0.759	0.93 [0.58, 1.48] 0.747	1.03 [0.62, 1.70] 0.913
*P* for trend	0.840	0.823	0.810
Bowel leakage of solid stool			
Iron intake (mg/day)			
Q1 (<9.95)	Ref	Ref	Ref
Q2 (9.95–13.45)	0.95 [0.53, 1.72] 0.866	1.01 [0.55, 1.87] 0.961	1.07 [0.56, 2.05] 0.829
Q3 (13.45–18.36)	1.34 [0.66, 2.70] 0.407	1.44 [0.69, 3.01] 0.310	1.55 [0.73, 3.30] 0.233
Q4 (>18.36)	1.28 [0.71, 2.30] 0.398	1.40 [0.76, 2.56] 0.268	1.47 [0.79, 2.76] 0.208
*P* for trend	0.258	0.168	0.123
Serum iron (umol/L)			
Q1 (<11.1)			
Q2 (11.1–14.5)	0.66 [0.38, 1.16] 0.142	0.67 [0.38, 1.19] 0.161	0.70 [0.38, 1.29] 0.232
Q3 (14.5–18.8)	0.88 [0.58, 1.32] 0.516	0.91 [0.61, 1.36] 0.627	0.93 [0.61, 1.42] 0.703
Q4 (>18.8)	**0.42 [0.20, 0.87] 0.021**	**0.43 [0.20, 0.91] 0.029**	**0.42 [0.20, 0.89] 0.027**
*P* for trend	**0.030**	**0.043**	**0.033**

OR: Odds Ratio, CI: Confidence Interval; Bolded values indicate statistically significant associations (*P* < 0.05).

As shown in Table 2, serum iron concentrations are inversely correlated with solid fecal leakage. In comparison to the first quartile, the fourth quartile of serum iron was linked to a 58% (model 1), 57% (model 2), and 58% (model 3) decreased risk of leaky bowel. The trend test was significant (P for trend <0.05), suggesting that the risk of leaky stool was reduced by 58% for each quartile increase in serum.

### 3.3 Restricted cubic splines

**Figure 2:**
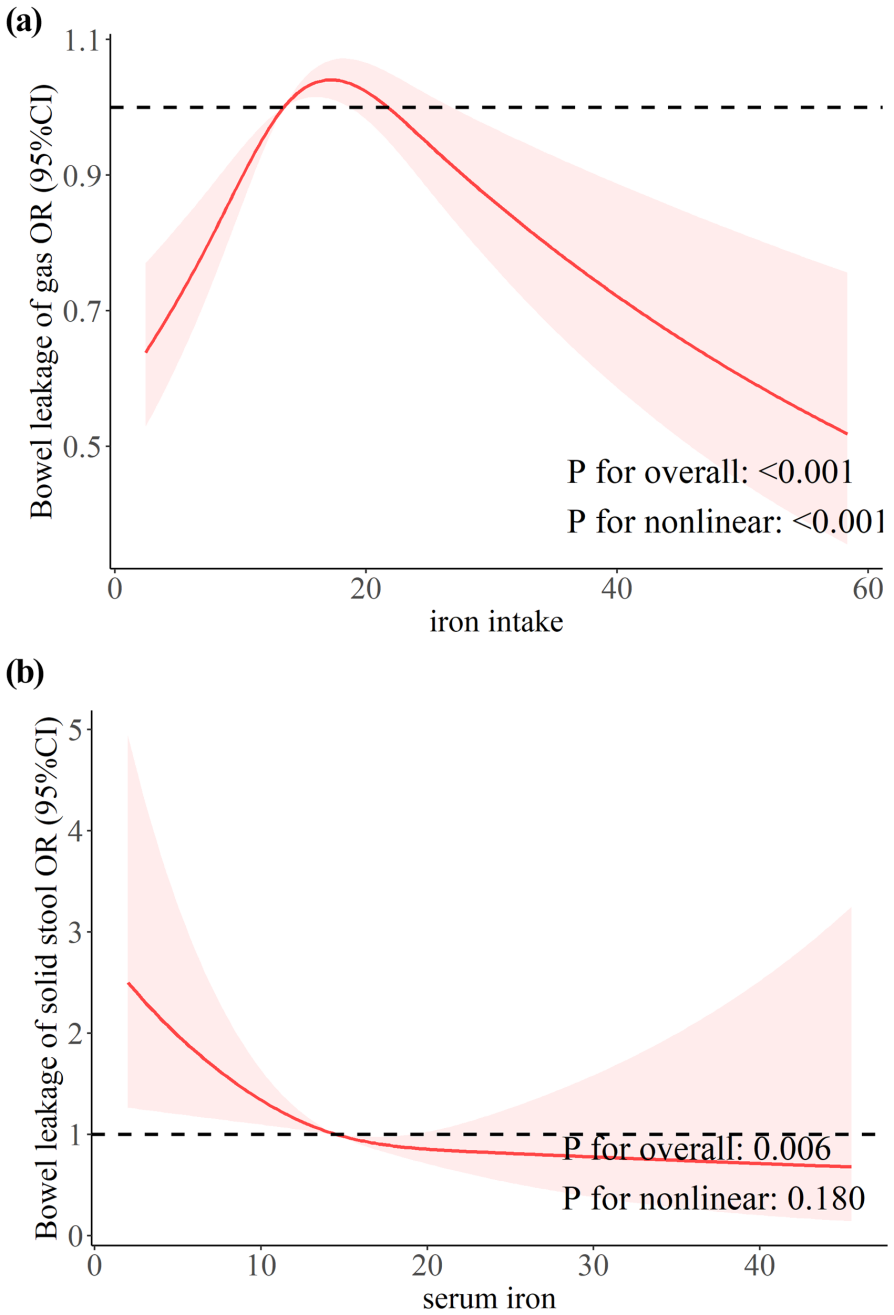
The dose–response relationship between dietary and serum iron with bowel leakage. **(a)** Dose–response relationship between dietary iron intake and bowel leakage of gas. **(b)** Dose–response relationship between serum iron and bowel leakage of solid stool.

Restricted cubic splines (RCS) were used to analyze the dose–response relationship between iron (dietary iron and serum iron) and leaky gut. As shown in Figure 2a, the weighted RCS results showed an inverse U-shaped association between iron intake and gas FI (*P* for nonlinear <0.001). The risk of gas FI increased when iron intake was between 13.68–21.55 mg/day. This parabolic downward trend suggests that high intake may have a protective effect. Conversely, serum iron concentrations were significantly negatively linearly (*P* for nonlinear = 0.180) correlated with the risk of solid stool leakage (Figure 2b). When the serum iron concentration exceeded 25umol/L, the comprehensive effect on solid stool leakage tended to be stable.

### 3.4 Subgroup analysis

#### 3.4.1 Subgroup analysis of dietary and serum iron with FI

The association heterogeneity of iron, gas, and solid stool leakage in different sociodemographic groups was further analyzed. As shown in Table 3, the association between dietary iron and bowel leakage was not significant in most subgroups. However, among non-Hispanic Black people, older women (60–74 years), and those with lower levels of education (below college), dietary iron was positively correlated with intestinal leakage risk. Moreover, the risk of solid bowel leakage was negatively correlated with serum iron levels in older adults, women, non-Hispanic White, those with low levels of education, those living in poverty, and those with a history of smoking, drinking, being overweight, or having high blood pressure. However, there was no statistically significant effect in the diabetic population.

**Table 3:** Subgroup analysis of dietary and serum iron with FI.

		Dietary iron and bowel leakage of gas			Serum iron and bowel leakage of solid stool		
Variables	*n* (%)	OR (95%CI)	*p*	*P* for interaction	OR (95%CI)	*p*	*P* for interaction
All patients	8,612 (100.00)	1.00 (1.00~1.01)	0.212		0.97 (0.95~1.00)	**0.030**	
Age				0.087			0.748
<45	3,422 (39.74)	1.00 (0.99~1.01)	0.887		0.99 (0.92~1.07)	0.835	
>74	970 (11.26)	1.02 (1.00~1.04)	0.064		0.97 (0.92~1.02)	0.260	
45–59	2,143 (24.88)	1.00 (0.99~1.01)	0.581		0.98 (0.94~1.03)	0.481	
60–74	2,077 (24.12)	1.01 (1.00~1.03)	**0.047**		0.95 (0.91~1.00)	**0.039**	
Gender				0.098			0.164
Female	4,428 (51.42)	1.01 (1.00~1.02)	**0.049**		0.95 (0.92~0.99)	**0.012**	
Male	4,184 (48.58)	1.00 (0.99~1.01)	0.886		0.99 (0.96~1.03)	0.647	
Race				0.055			0.074
Non-Hispanic Black	1,518 (17.63)	1.02 (1.00~1.03)	**0.028**		1.00 (0.94~1.07)	0.896	
Non-Hispanic White	4,369 (50.73)	1.00 (0.99~1.01)	0.962		0.95 (0.92~0.98)	**0.004**	
Other	2,725 (31.64)	1.00 (0.99~1.01)	0.488		1.00 (0.95~1.05)	0.913	
Education				0.077			0.450
Below college	4,434 (51.49)	1.01 (1.00~1.02)	**0.024**		0.96 (0.93~0.99)	**0.024**	
College or above	4,178 (48.51)	1.00 (0.99~1.01)	0.637		0.99 (0.95~1.03)	0.495	
PIR				0.660			0.168
Not poor	4,905 (56.96)	1.01 (1.00~1.01)	0.157		0.99 (0.95~1.02)	0.439	
Poor	3,707 (43.04)	1.00 (0.99~1.01)	0.754		0.95 (0.92~0.99)	**0.019**	
BMI				0.546			0.638
Normal	2,406 (27.94)	1.00 (0.99~1.01)	0.844		0.98 (0.94~1.02)	0.262	
Obese	3,249 (37.73)	1.00 (0.99~1.01)	0.432		0.99 (0.94~1.04)	0.655	
Overweight	2,957 (34.34)	1.01 (1.00~1.02)	0.092		0.95 (0.90~1.00)	**0.041**	
Alcohol				0.407			0.162
No	2,398 (27.84)	1.01 (1.00 ~ 1.02)	0.133		1.00 (0.95~1.04)	0.924	
Yes	6,214 (72.16)	1.00 (1.00 ~ 1.01)	0.541		0.96 (0.93~0.99)	**0.011**	
Smoke				0.629			0.568
Former smoker	2,248 (26.10)	1.01 (1.00~1.02)	0.094		0.95 (0.91~1.00)	**0.037**	
Never smoke	4,561 (52.96)	1.00 (0.99~1.01)	0.547		0.99 (0.95~1.04)	0.783	
Recent smoker	1,803 (20.94)	1.00 (0.99~1.01)	0.804		0.95 (0.90~1.01)	0.083	
Hypertension				0.407			0.248
No	5,517 (64.06)	1.00 (1.00~1.01)	0.517		0.99 (0.95~1.02)	0.520	
Yes	3,095 (35.94)	1.01 (1.00~1.02)	0.212		0.95 (0.92~0.99)	**0.013**	
Diabetes				0.165			0.468
No	7,151 (83.04)	1.00 (1.00~1.01)	0.513		0.97 (0.94~1.00)	**0.025**	
Yes	1,461 (16.96)	1.01 (1.00~1.03)	0.085		0.98 (0.93~1.03)	0.518	
Depression				0.876			0.421
No	7,802 (90.59)	1.00 (1.00~1.01)	0.252		0.98 (0.95~1.00)	0.095	
Yes	810 (9.41)	1.01 (0.99~1.02)	0.591		0.95 (0.88~1.01)	**0.114**	

OR, Odds ratio; CI, Confidence interval; Bolded values indicate statistically significant associations (*P* < 0.05).

#### 3.4.2 Exploratory biomarker analysis

We attempted to explore the association of total iron-binding capacity and unsaturated iron-binding capacity with FI, but the NHANES 2007–2010 cycle did not include these data. Whereas, NHANES mainly collects ferritin and transferrin receptor levels in women of childbearing age. Therefore, we performed additional analyses of serum ferritin and transferrin receptors in a subset of female participants aged 20–49 years (*n* = 2,202), as these biomarkers were only measured in participants aged 1–5 years, as well as in women aged 12–49 years, while participants who completed the bowel health questionnaire were all over 20 years of age. In this subgroup, the highest quartile of ferritin (OR = 0.30, 95% CI 0.11–0.83) was significantly associated with mucus FI after full adjustment (Supplementary Table S2). The results of the trend test (*P* for trend = 0.004) showed that the risk of mucus FI gradually decreased with the increase of ferritin levels.

## 4 Discussion

This is the first study to show that dietary iron and serum iron have a synergistic effect on FI using a nationally representative sample. Firstly, serum iron was negatively linearly associated with solid FI. Secondly, there was a nonlinear, inverted U-shaped relationship between iron intake and gas gut leakage. This association between iron and FI is most pronounced in older people, women, and groups with low levels of education. Lastly, in women of childbearing age between 20 and 49, ferritin levels were negatively correlated with mucus gut leakage. In addition, no association was found between iron and liquid gut leakage.

Our findings suggested that in the adult population, iron intake between 13.68 and 21.55 mg/day was associated with an increased risk of gas FI. At the same time, lower and higher doses did not show a statistically significant association with FI. Beneficial bacteria with low iron needs, such as Lactobacilli and Bifidobacteria, were more prevalent and could prevent pathogen colonization when iron intake is low (29–31). Thus, at lower levels, iron consumption did not raise the risk of FI. As iron intake increased, iron-loving pathogens such as *E. coli*, Salmonella, and Heraldella predominated, promoting intestinal inflammation and increasing diarrhea (32–34). Besides, diarrhea was a known risk factor for FI, and iron supplementation had also been shown to increase intestinal permeability (35, 36). Therefore, by promoting intestinal inflammation and raising intestinal permeability, higher doses of iron consumption raised the risk of FI. Higher intake of iron may have a protective effect against FI. Several studies support our results. For example, a randomized controlled study of 53 Swedish infants aged 6 months reported a significantly higher abundance of beneficial bacteria in the intestines of infants in the high-iron group (6.6 mg Fe/day) compared with the low-iron group (1.2 mg Fe/day) (37). The iron intake of infants in both the low-iron group and the high-iron group exceeded the recommended intake (RDA) of 0.27 mg/d for infants aged 0–6 months stipulated by the National Institutes of Health (NIH) and the World Health Organization (WHO). A study of dietary nutrients in patients with FI and controls showed that iron intake was higher in the non-FI group. The iron intake in the FI group was 119 ± 45% of the RDA, while the iron intake in the control group was 128 ± 51% of the RDA (38). In addition, an animal study showed that high doses of iron can promote intestinal repair by modulating intestinal epithelial cell turnover and intestinal stem cell activity (39). According to a study by Sarah R. Bloor et al., too much iron intake damages intestinal villi and impairs the intestinal barrier, which in turn causes intestinal inflammation (40, 41). The latter, in turn, triggers the production and secretion of hepcidin, which lowers the levels of the divalent metal transporter 1 (DMT-1) and reduces iron absorption (41). Therefore, different levels of dietary iron intake may increase the risk of FI by altering the ratio of beneficial and pathogenic bacteria in the gut or altering intestinal permeability. High iron intake protected the intestines by upregulating hepcidin and promoting intestinal repair.

This appeared to challenge previous theories about the harmful effects of iron overload on the gut. In an animal experiment, for instance, mice fed a high-iron diet containing 1,000 mg per day for 70 days had a significant decrease in Cldn8, a marker of intestinal barrier integrity, which led to luminal bacterial leakage, a decrease in beneficial bacteria like Tanner and Ekmania, and a significant enrichment of pathogenic bacteria like Streptococcus peptococcus (42). In addition, the study by Tanja Jaeggi et al. reported that iron fortification (12.5 mg/day, WHO recommends 0.27 mg/day for infants) adversely affected the gut microbiome of Kenyan infants, increasing pathogen abundance and inducing intestinal inflammation (43). The contradiction between our study and previous research could be explained by the following points. A recent review of iron and gut microbiota showed that multiple studies reported different or even opposite results due to differences in the models and detection methods used – except for the Lactobacillus family, which always decreased during iron supplementation, the gut microbiota (Bifidobacteria, Bacteroides) of the other species showed different or even contradictory alterations (44). Therefore, the mechanism of iron’s effect on the gut microbiota is complex and variable. Besides, while most of these studies were conducted through short, high-dose iron supplementation, our study was based on the daily iron intake of adults, which was much lower than the amount of iron in the experiments. In addition, different forms of iron supplementation had different effects on gut microbiota, and there were no studies on daily iron intake and gut microbiota (31).

Similarly, in this study, the majority of patients with FI were elderly and female, which was consistent with the results of previous studies (45, 46). This might be related to aging increasing colonic transit time and decreasing anal sphincter pressure (47, 48).

We found that higher serum iron levels were associated with a lower risk of solid FI. Prior research showed a negative correlation between serum iron and factors that are overexpressed in inflammatory and tumor situations, such as growth differentiation factor-15 (GDF-15) and IL-6 (49, 50). It has been reported that iron intake can increase serum iron levels, regulate goblet cell regeneration and mucin layer function, and play a positive role in the prevention of pathogenic bacteria (51). In addition, subgroup analysis showed that this association between serum iron and FI was more significant in women, older adults, poor (PIR < 2), smokers, alcohol users, and hypertension, but not in people with diabetes. These results suggested that the protective effect of serum iron might be co-regulated by aging-related metabolic remodeling, differences in the regulation of oxidative stress, and health behavior-nutrition interactions. Diabetes-related pathological mechanisms might negate its potential benefits.

Although our analysis of serum ferritin was limited to women of reproductive age, given the restrictions of NHANES data, our study demonstrated a significant inverse relationship between serum ferritin levels and mucus FI. A previous study showed that increased ferritin concentrations appeared to be associated with a reduced risk of colorectal cancer, but the results were not significant (20). In addition, systemic immune inflammation was significantly negatively correlated with serum ferritin (52). This seemed to support our results. However, some studies have pointed out that ferritin levels are negatively correlated with beneficial bacteria such as Bifidobacteria and lactic acid bacteria, but positively correlated with conditionally pathogenic bacteria such as Bacteroides and Prevonella (53). The association of ferritin with FI might be related to gut microbiota and inflammation, but further study is required to validate this.

A study on the effects of iron intake on mice with Crohn’s disease suggested that an iron-deficient diet might suppress intestinal inflammation (54), however, several studies showed that iron overload or iron deficiency might impair gut health by affecting the gut microbiota. In our research, the risk of gas FI increased when iron intake was 13.68–21.55 mg/day. Therefore, consuming less or more iron might be a better option for FI. Previous studies showed that iron deficiency was associated with intestinal diseases such as inflammatory bowel disease. In addition, the WHO stated that the maximum safe daily intake (UL) for iron was 45 mg/day, and exceeding this dose might lead to health risks. Therefore, we recommended consuming as much iron as possible at a safe dose, i.e., 22–45 mg/day. Besides, serum iron and ferritin were found to be inversely associated with solid/mucus FI. Higher iron intake increased the levels of serum iron and ferritin, along with a reduced risk of solid FI and mucus FI. Therefore, higher iron intake in safe doses might be beneficial in reducing the risk of FI.

There are several advantages to our research. Firstly, our study uses nationally representative data from NHANES and applies the weights recommended by NHANES to improve generalization and generalization of results. Besides, our study adjusts for confounding factors such as demographics, social factors, chronic diseases, etc. In addition, previous studies have focused on the linear effects of iron deficiency or iron overload, while our study further assess the dose-effect effect of iron on FI.

There are also limitations to our study. Firstly, underreporting of FI due to stigma may underestimate the true prevalence. Secondly, the cross-sectional design prevents causal inference. A combination of anal sphincter electromyography (EMG) and iron staining techniques may be needed in the future to verify a direct association between tissue iron and loss of function, or further prospective studies may be needed to illustrate this.

## 5 Conclusion

Iron intake of 13.68–21.55 mg/day nonlinearly increases gas FI risk, while higher serum iron linearly reduces solid FI. Associations are strongest in women and older adults. Optimizing iron balance—avoiding moderate dietary excess and ensuring serum sufficiency—may prevent FI, particularly in high-risk groups.

## Data Availability

The datasets presented in this study can be found in online repositories. The names of the repository/repositories and accession number(s) can be found in the article/Supplementary material.

## Footnotes

^1^ https://wwwn.cdc.gov/nchs/nhanes/
