# Supplementary material for: The association of dietary iron intake and serum iron with fecal incontinence: results from NHANES 2007–2010
Source: Front Nutr. 2025 Jun 19;12:1598172. doi: 10.3389/fnut.2025.1598172 (PMC12224654; doi:10.3389/fnut.2025.1598172)
Supplement: Supplementary file 1 [file Table_1.docx]

Supplementary Material

**Table S1: Explanation and classification of covariates**

|  | **Covariates** | **Explanation and classification** |
| --- | --- | --- |
| Demographics | Age | Quantitative data |
|  | Gender | Female; Male |
|  | Race | Non-Hispanic Black; Non-Hispanic White; Other |
|  | Education | Below college; college and above |
|  | PIR | The ratio of family income to poverty. |
|  | BMI | Body mass index. Overweight is defined as a BMI<25kg/m^2^, obesity is defined as a BMI≥30 kg/m^2^, with a BMI of 25-30 kg/m^2^ being normal. |
| Lifestyle | Smoke | Recent smoker: People who answer "every day" or "some days" to "Do you now smoke cigarettes" are considered **recent smoker**. **Former smoker**: People who have smoked at least 100 cigarettes before, but not smoke now. **Never smoke**: People who do not smoke now and have not smoked 100 cigarettes in their lifetime. |
|  | Alcohol | It is determined by whether you have drunk at least 12 glasses of alcohol in your life. |
| Comorbidities | Hypertension | People who have been told to have high blood pressure. |
|  | Diabetes | People who have been told by health care providers to have diabetes, use hypoglycemic drugs, insulin injections, or related abnormalities (Glycosylated hemoglobin ≥ 6.5% or fasting blood glucose ≥ 126 mg/dL) are considered to have diabetes. |
|  | Depression | A PHQ-9 score of more than 10 is thought to be significantly associated with a depressive state. |

**Table S2: Association of ferritin and transferrin receptors with FI in a subgroup of women of childbearing age**

| Item | Model 1  OR (95%CI) P value | Model 2  OR (95%CI) P value | Model 3  OR (95%CI) P value |
| --- | --- | --- | --- |
| **Bowel leakage of gas** |  |  |  |
| 1. Ferritin (ug/L) |  |  |  |
| Q1 (<20.1) | Ref | Ref | Ref |
| Q2 (20.1-38.0) | 1.05 [0.76, 1.46] 0.752 | 1.02 [0.74, 1.41] 0.878 | 1.03 [0.73, 1.45] 0.858 |
| Q3 (38.0-69.0) | 1.07 [0.74, 1.53] 0.723 | 1.03 [0.71, 1.49] 0.873 | 1.03 [0.70, 1.50] 0.886 |
| Q4 (>69.0) | 1.01 [0.68, 1.51] 0.958 | 0.99 [0.66, 1.46] 0.942 | 0.98 [0.65, 1.46] 0.895 |
| P for trend | 0.936 | 0.841 | 0.780 |
| 2. Transferrin receptor (mg/L) | 1.00 [0.96, 1.05] 0.931 | 1.02 [0.97, 1.07] 0.419 | 1.01 [0.97, 1.06] 0.546 |
| P for trend | 0.319 | 0.116 | 0.201 |
| **Bowel leakage of mucus** |  |  |  |
| 1. Ferritin (ug/L) |  |  |  |
| Q1 (<20.1) | Ref | Ref | Ref |
| Q2 (20.1-38.0) | 1.24 [0.41, 3.73] 0.697 | 1.32 [0.43, 4.05] 0.615 | 1.41 [0.47, 4.19] 0.519 |
| Q3 (38.0-69.0) | 0.77 [0.27, 2.15] 0.604 | 0.79 [0.28, 2.22] 0.645 | 0.79 [0.28, 2.22] 0.633 |
| Q4 (>69.0) | **0.37 [0.14, 0.98] 0.046** | 0.38 [0.14, 1.07] 0.067 | **0.30 [0.11, 0.83] 0.023** |
| P for trend | **0.014** | **0.022** | **0.004** |
| 2. Transferrin receptor (mg/L) | 1.00 [0.90, 1.11] 0.977 | 1.01 [0.89, 1.16] 0.837 | 0.98 [0.82, 1.18] 0.837 |
| P for trend | 0.237 | 0.197 | 0.373 |
| **Bowel leakage of liquid** |  |  |  |
| 1. Ferritin (ug/L) |  |  |  |
| Q1 (<20.1) | Ref | Ref | Ref |
| Q2 (20.1-38.0) | 1.04 [0.37, 2.95] 0.937 | 1.07 [0.37, 3.09] 0.896 | 1.22 [0.40, 3.72] 0.705 |
| Q3 (38.0-69.0) | 1.00 [0.33, 3.03] 0.997 | 1.02 [0.33, 3.17] 0.977 | 1.12 [0.35, 3.55] 0.839 |
| Q4 (>69.0) | 1.13 [0.40, 3.24] 0.806 | 1.16 [0.40, 3.39] 0.771 | 1.22 [0.45, 3.30] 0.673 |
| P for trend | 0.774 | 0.743 | 0.679 |
| 2. Transferrin receptor (mg/L) | 0.97 [0.88, 1.06] 0.462 | 0.98 [0.89, 1.08] 0.623 | 0.95 [0.84, 1.06] 0.329 |
| P for trend | 0.199 | 0.131 | 0.279 |
| **Bowel leakage of solid stool** |  |  |  |
| 1. Ferritin (ug/L) |  |  |  |
| Q1 (<20.1) | Ref | Ref | Ref |
| Q2 (20.1-38.0) | 0.66 [0.11, 3.99] 0.640 | 0.64 [0.09, 4.38] 0.635 | 0.72 [0.09, 5.66] 0.743 |
| Q3 (38.0-69.0) | 0.97 [0.19, 4.84] 0.968 | 0.89 [0.15, 5.49] 0.899 | 1.00 [0.16, 6.37] 0.999 |
| Q4 (>69.0) | 0.36 [0.06, 2.00] 0.231 | 0.33 [0.05, 2.14] 0.231 | 0.34 [0.05, 2.28] 0.249 |
| P for trend | 0.348 | 0.341 | 0.370 |
| 2. Transferrin receptor (mg/L) | 1.07 [1.01, 1.13] 0.033 | 1.06 [0.99, 1.14] 0.070 | 1.06 [0.99, 1.14] 0.105 |
| P for trend | **0.043** | **0.039** | 0.058 |
